# The temperature sensor TWA1 is required for thermotolerance in *Arabidopsis*

**DOI:** 10.1038/s41586-024-07424-x

**Published:** 2024-05-15

**Authors:** Lisa Bohn, Jin Huang, Susan Weidig, Zhenyu Yang, Christoph Heidersberger, Bernard Genty, Pascal Falter-Braun, Alexander Christmann, Erwin Grill

**Affiliations:** 1 Chair of Botany, TUM School of Life Sciences Weihenstephan, Technische Universität München (TUM), Freising, Germany; 2 Aix-Marseille University, Commissariat à l’Energie Atomique (CEA), Centre National de la Recherche Scientifique (CNRS), Institut de Biosciences et Biotechnologies Aix-Marseille, Saint-Paul-lez-Durance, France; 3 Institute of Network Biology (INET), Molecular Targets and Therapeutics Center (MTTC), Helmholtz Center Munich, German Research Center for Environmental Health, Munich, Germany; 4 Microbe-Host Interactions, Faculty of Biology, Ludwig-Maximilians-Universität (LMU) München, Munich, Germany; 5 Present Address: Chengdu Newsun Crop Science, Chengdu, China

**Keywords:** Heat, Plant hormones, Transcription

## Abstract

Plants exposed to incidences of excessive temperatures activate heat-stress responses to cope with the physiological challenge and stimulate long-term acclimation^1,2^. The mechanism that senses cellular temperature for inducing thermotolerance is still unclear^3^. Here we show that TWA1 is a temperature-sensing transcriptional co-regulator that is needed for basal and acquired thermotolerance in *Arabidopsis thaliana*. At elevated temperatures, TWA1 changes its conformation and allows physical interaction with JASMONATE-ASSOCIATED MYC-LIKE (JAM) transcription factors and TOPLESS (TPL) and TOPLESS-RELATED (TPR) proteins for repressor complex assembly. TWA1 is a predicted intrinsically disordered protein that has a key thermosensory role functioning through an amino-terminal highly variable region. At elevated temperatures, TWA1 accumulates in nuclear subdomains, and physical interactions with JAM2 and TPL appear to be restricted to these nuclear subdomains. The transcriptional upregulation of the heat shock transcription factor A2 (HSFA2) and heat shock proteins depended on TWA1, and TWA1 orthologues provided different temperature thresholds, consistent with the sensor function in early signalling of heat stress. The identification of the plant thermosensors offers a molecular tool for adjusting thermal acclimation responses of crops by breeding and biotechnology, and a sensitive temperature switch for thermogenetics.

## Abstract (web summary)

TWA1 is a temperature-sensing transcriptional co-regulator that is needed for basal and acquired thermotolerance in *Arabidopsis thaliana.*

## Main

Climatic temperature gradients constrain growth and fitness of plants. Plant species have preferred temperatures with daytime optima that vary substantially from 10 °C for the cool-preferring viola to 35 °C for the tropical kapok tree^4^. Temperatures substantially exceeding the optimum trigger acclimation responses that involve development and the heat-stress response (HSR)^2^. Several plant thermosensors that regulate temperature-induced developmental processes are known, such as Ca^2+^channels, transcriptional regulators and light receptors^5,6^.

In metazoans, fungi and plants, heat triggers the HSR through HSF1 transcription factors (TFs)^1,7^. After release from inhibitory molecular chaperones^8^, HSF1 induces expression of heat-shock proteins (HSPs) and initiates adaptation. In plants, the HSR involves several phytohormone pathways^9–13^. *A. thaliana* mutants that are deficient in biosynthesis or insensitive to the phytohormone abscisic acid (ABA) are compromised in basal and acquired thermotolerance^14^.

ABA is the key signal to regulate the water status of plants by adjusting transpiration and water resorption to the demand of atmospheric CO_2_ for photosynthesis^15,16^. Under water deficit, ABA levels increase and induce protective measures including stomatal closing, differential gene expression, downregulation of chlorophylls and biosynthesis of osmolytes such as proline. In natural environments, heat and drought occur often combined and are the cause of major crop losses^17^. Stimulating ABA signalling through ABA receptors can reduce transpiration while sustaining photosynthesis and growth, therefore offering an option for a more water-efficient agriculture^18^. However, reduced transpiration increases leaf temperature and may thereby acerbate thermal stress during a heat wave. On the other hand, ABA promotes thermotolerance, and enhanced ABA signalling might be beneficial for heat acclimation.

## Identification of TWA1

**Fig. 1:**
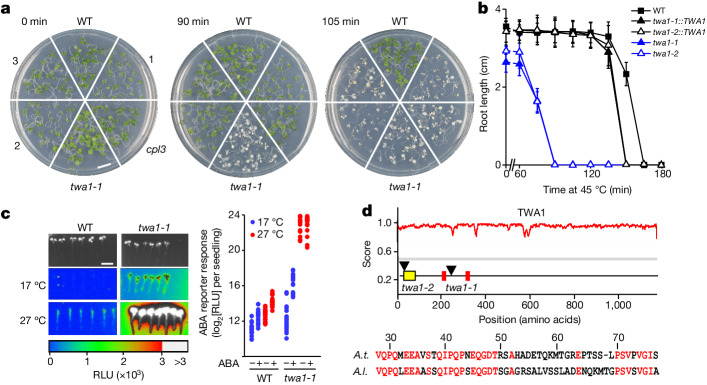
The *TWA1* locus. **a**, The *A. thaliana twa1-1* mutant is conspicuously thermosensitive. Five-day-old seedlings of WT and mutants with ABA-hypersensitive gene regulation were exposed to 45 °C for 90 or 105 min or not after 90 min priming at 38 °C. Pictures were taken 5 days after heat stress. **b**, The acquired thermotolerance of WT, *twa1-1* and *twa1-2* plants, as well as mutants complemented with the *TWA1* gene, that were assayed for root growth within 5 days after heat exposure as indicated. **c**, ABA-inducible *pHB6:LUC* expression in 5-day-old seedlings exposed to 1 µM ABA for 16 h at 17 °C or 27 °C. Left, photographs and light emission of WT and *twa1-1* plants expressing the reporter. Right, reporter activity per seedling given in relative light units (RLU; *n* = 18). **d**, Prediction of intrinsically disordered regions (https://metapredict.net/) with maximum score of 1, and a schematic of TWA1, showing the highly variable region (HVR, yellow) and two EAR domains (red) (top). TWA1 disruption in mutants is shown by arrowheads. Bottom, amino acid identity (red) of the HVR from *A. thaliana* (*A.t*.) and *A. lyrata* (*A.l*.). For **b**, data are mean ± s.d.; *n* = 6 per datapoint. Experimental and statistical significance details are provided in the Methods and as Source Data. Source Data

**Extended Data Fig. 1:**
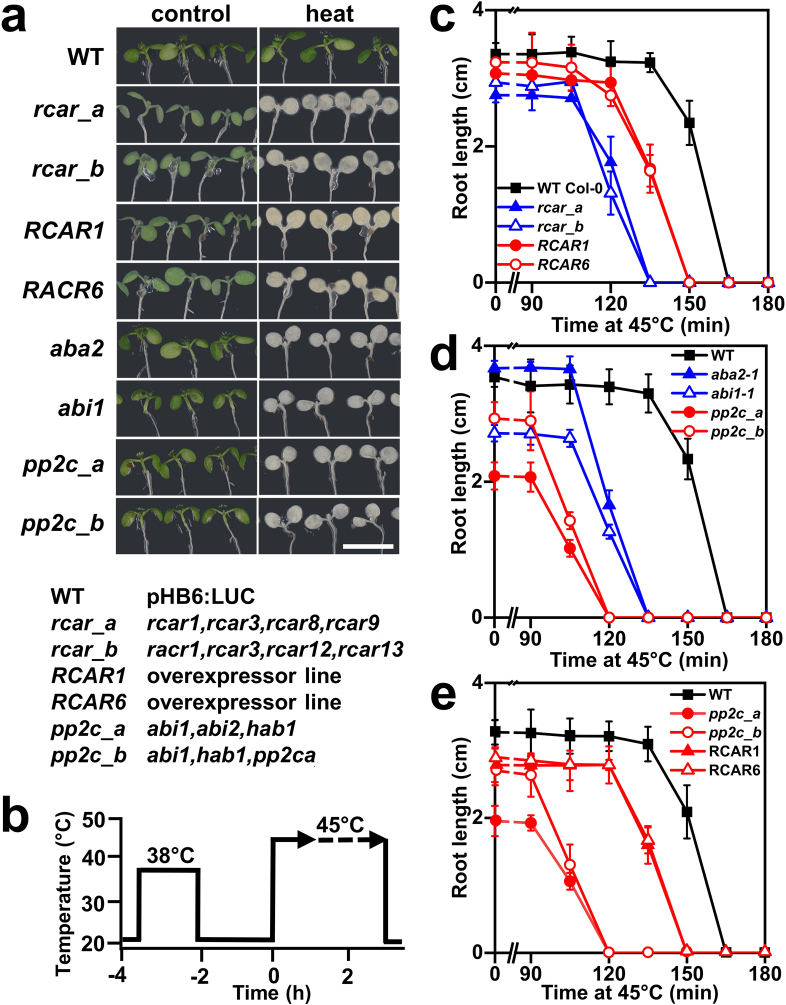
Seedlings with enhanced ABA signalling are impaired in acquired thermotolerance. **a**, Phenotypes of 5-day-old seedlings assayed for acquired tolerance. Pictures of wild type and lines with altered ABA response were taken one day after or without 150 min heat stress at 45 °C. Scale bar depicts 1 cm. **b**, Scheme of heat exposures in **a**. **c, d**, Inhibition of root growth in lines with reduced (blue) and increased ABA response (red) compared to WT (reporter line *pHB6:LUC* in Col-0 accession (**d**), or Col-0 (**c**)) exposed to 45 °C for different times. Inhibition was determined 5 days after stress. **e**, Inhibition of root growth by heat stress in the dark. 4 day-old acclimated seedlings of wild type, mutants *pp2c_a* and *pp2c_b*, and RCAR1 and RCAR6 overexpressing lines were exposed to 45 °C heat stress for the indicated times in darkness. Root extension was measured 5 days after treatment. **c-e**, Mean ± s.d. (n = 6). Statistical significance and source data are provided in the Supplementary Information (SI) File Extended Data Fig. 1. Source Data

**Extended Data Fig. 2:**
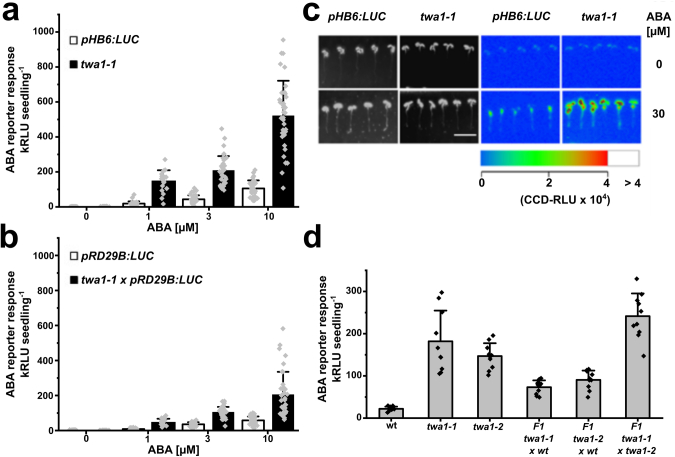
Hypersensitive ABA reporter response of twa1 mutants. **a**, Induction of luciferase activity by exogenous ABA in wild type reporter line *pHB6:LUC* and *twa1-1* mutant of *pHB6:LUC* genotype. 5-day-old seedlings were exposed to ABA for 24 h before luminescence measurements (mean ± SD, n = 36 per data point). **b**, ABA-induced luciferase in wild type *pRD29B:LUC* reporter line and *twa1-1* mutant in the *pRD29B:LUC* genotype analysed as in **a**. **c**, Life imaging of *pHB6:LUC* or *twa1-1* seedlings exposed to no and 30 µM ABA. Bright field and light emission photographs in false colours for relative light units (CCD-RLU) per seedling. Scale bar: 1 cm. **d**, Partial haplo-insufficiency of *twa1-1* and *twa1-2* alleles. F1 crosses and lines homozygous for the reporter were exposed to 3 µM ABA for 24 h (mean ± SD, n = 10). Statistical significance in SI_Extended Fig. 2. Source Data

**Extended Data Fig. 3:**
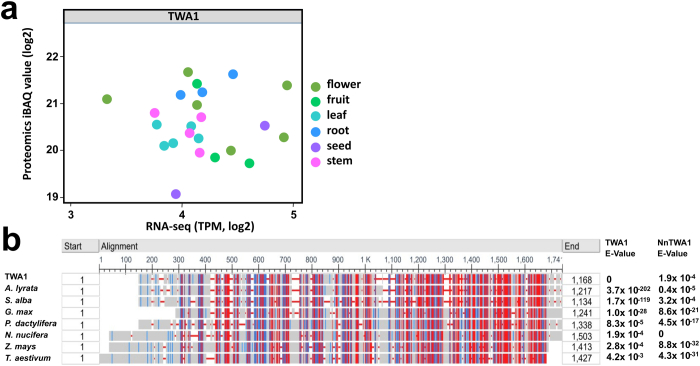
TWA1 expression and TWA1 homologues. **a**, TWA protein and *TWA1* transcript abundance according to ATHENA proteomics database (https://athena.proteomics.wzw.tum.de)^25^. TPM, transcripts per kilobase and million reads; iBAQ are summed peptide intensity per protein normalized to the number of expected peptides. **b**, Alignment of TWA1 homologues identified by BLASTp searches against plant genomes on NCBI. Amino acid sequences were aligned with NCBI’s COBALT^71^. Alignments in red indicate highly conserved regions and lower conservation is shown in blue. E-values were calculated by using FASTA (https://fasta.bioch.virginia.edu) with TWA1 and lotus NnTWA1 as reference. Sequences: TWA1 - NP_001331741.1, *Arabidopsis lyrata* - XP_002873619.1, *Sinapis alba* - AF8055519.1, *Glycine max* - KAG5128726.1, *Nelumbo nucifera* - A0A1U7ZPG7 and from monocots: *Phoenix dactylifera* - A0A8B7BGH8, *Zea mays* - XP_035823187.1, *Triticum aestivum* - XP_044429385.1.

To better understand the role of ABA in heat tolerance, *A. thaliana* lines that are hyper-responsive or insensitive towards ABA were examined for alterations in acquired thermotolerance induced by a priming heat exposure. Ectopic expression of ABA receptors RCAR6 and RCAR1 or deficiency in ABA co-receptors encoded by type 2C protein phosphatases (PP2Cs) results in ABA hypersensitivity^18,19^, while deficiency in multiple ABA receptors leads to ABA hyposensitivity^20,21^. Seedlings with such changes in the ABA response, including previously analysed ABA mutants^14^, were exposed to a priming period at 38 °C followed by a recovery phase and a subsequent variable period of heat stress at 45 °C (Extended Data Fig. 1a,b). Both, ABA-hypersensitive and ABA-insensitive lines were more susceptible to heat stress compared with the wild type (WT; Extended Data Fig. 1a,c,d). The ABA-hypersensitive lines had a heat-sensitive phenotype irrespective of light (Extended Data Fig. 1e). The findings argue for a dual role of ABA during heat stress, that is, initially promoting thermotolerance but eventually being counterproductive for the acclimation process. ABA receptors and PP2C co-receptors control fast and slow responses including ion channels and aquaporins by fast post-translational control and differential gene expression for long-term adjustments^15,16^. ABA hypersensitivity at the level of gene expression is conferred by deficiency in the C-terminal domain phosphatase-like (CPL) family members such as CPL3^22^. We examined the thermotolerance of the *cpl3*-knockout mutant and four additional mutants identified in a screen for ABA hypersensitivity using an ABA-responsive luciferase (LUC) reporter line^23^ (Methods). All of these mutants were more heat sensitive than the WT line (Fig. 1a). One mutant line showed a strongly enhanced thermosensitivity with a half-maximum inhibitory period (IP_50_) of approximately 75 min at 45 °C, whereas the *pp2c* triple mutant and WT lines had IP_50_ values of 105 min and 155 min, respectively (Fig. 1b and Extended Data Fig. 1d). The gene locus was named *THERMO-WITH ABA-RESPONSE 1* (*TWA1*). In the presence of exogenous ABA, the induction of ABA-responsive LUC reporters was enhanced in the *twa1-1* background (Fig. 1c and Extended Data Fig. 2a–c), indicating hyperactivation of ABA signalling. Shifting the temperature from 17 °C to 27 °C induced a 3-fold increase in reporter expression in WT plants but caused a more than 100-fold increase in the *twa1-1* seedlings (Fig. 1c). The *twa1-1* mutant showed partial haploinsufficiency in a cross with the WT (Extended Data Fig. 2d) and the affected gene (*At5g13590*) was identified (Methods). The *TWA1* locus encodes a predicted 130 kDa intrinsically disordered protein of unknown function with two potential ethylene-responsive-element-binding-factor-associated amphiphilic repression (EAR) motifs (LxLxL)^24^ (Fig. 1d). A point mutation in the *twa1-1* allele terminates translation after 247 amino acids. The transfer DNA (T-DNA) knockout line *twa1-2* with disrupted *TWA1* in the first exon was heat sensitive, comparable to *twa1-1* (Fig. 1b). Transfer of a genomic fragment encompassing the *TWA1* gene complemented the thermosensitivity of both mutants (Fig. 1b). TWA1 is expressed throughout the plant^25^ (Extended Data Fig. 3a).

## Thermosensor function

**Fig. 2:**
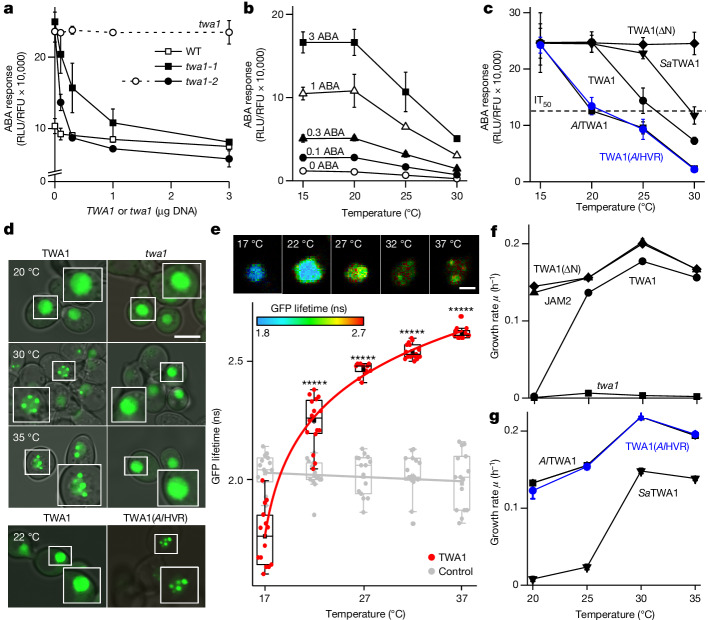
The thermosensor function of TWA1. **a**–**c**, TWA1 and TWA1 variants inhibit ABA-responsive *pRD29B:LUC* expression in leaf protoplasts. **a**, Protoplasts of WT, *twa1-1* and *twa1-2* plants were transfected with a *TWA1*or *twa1-1* expression cassette (shown as µg DNA per 10^5^ protoplasts), incubated for 16 h at 25 °C with 10 µM ABA. Relative light units were normalized to fluorescence units of a co-transfected control (RFU). The dashed line indicates expression of mutant twa1 product in *twa1-2* protoplasts. **b**, TWA1 action at different exogenous ABA levels. **c**, The action of TWA1 and variants at different incubation temperatures with 0.3 µg effector DNA per 10^5^ *twa1-2* protoplasts. The variants included TWA1(ΔN), *A. lyrata* *Al*TWA1, mustard *Sa*TWA1 and TWA1(*Al*HVR). **d**, Top, GFP–tagged TWA1 and twa1 product in yeast nuclei and temperature-dependent phase separation. Bottom, nuclear subdomain formation of TWA1(*Al*HVR) at 22 °C. Insets: 1.5-fold magnifications. Scale bar, 10 μm. See the ‘Statistics and reproducibility’ section of the Methods. **e**, Temperature-induced changes in the proximity of TWA1 amino- and carboxy-terminal domains as revealed by intramolecular FRET–FLIM analysis. Top, false-colour images of GFP FLs of mCherry–TWA1–GFP (TWA1) in yeast nuclei. Scale bar, 2 µm. Bottom, GFP FL of TWA1 and mCherry–GFP. *n* = 10–15 per datapoint. Statistical analysis was performed using Student’s *t*-tests; *****P = 10^−5^. For the box plots, the centre line shows the median, the box limits show the upper (25th percentile) and lower (75th percentile) quartiles, and the whiskers show 1.5× the interquartile range. **f**,**g**, The temperature dependence of JAM2 binding to TWA1 and variants including the twa1 product (**f**) and binding to orthologues (**g**) was analysed using a Y2H growth assay. The growth rate *µ* was calculated on the basis of cell density increase within 24 h. JAM2–JAM2 dimerization served as the control (Extended Data Fig. 7). For **a**–**c**,**f**,**g**, *n* = 3 per datapoint. Statistical significance details are provided as Source Data. Source Data

**Extended Data Fig. 4:**
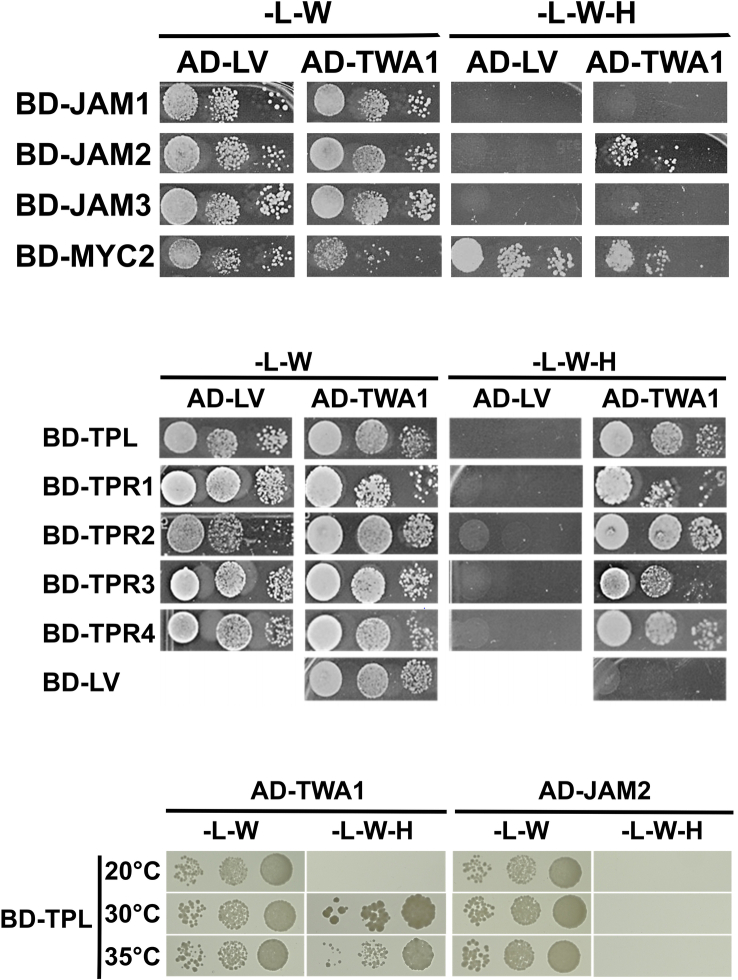
Yeast-Two-Hybrid analysis of TWA1-protein interaction. Top, Physical interaction of TWA1 with JAM transcription factors of *A. thaliana*. TWA1 fused to the GAL4-activation domain (AD) was assayed for interaction with JAMs/MYC2 fused to Gal 4-DNA binding domain (BD) at 30 °C. Middle, transcriptional corepressors TPL and TPRs bind to TWA1 at 30 °C. Bottom, the interaction of TPL with the thermosensor is temperature dependent. Yeasts were grown in the absence of leucine and tryptophan (-L-W), and with or without histidine (H) using serial tenfold culture dilutions. Controls are empty expression cassettes (LV).

**Extended Data Fig. 5:**
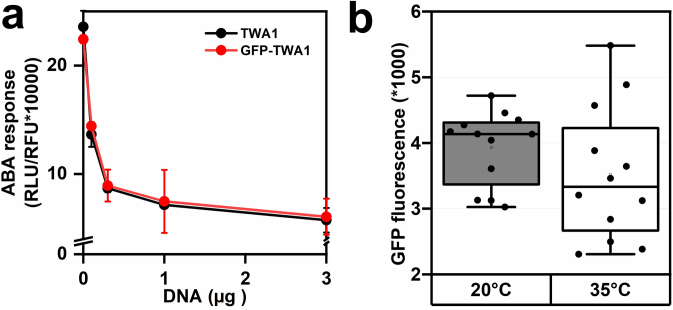
GFP-TWA1 is functional in protoplasts and GFP-TWA1 abundance in response to temperature in yeast. **a**, Capacity of TWA1 and GFP-TWA1 to inhibit ABA signalling in *twa1-2* protoplasts*. The* ABA-responsive luciferase reporter (*pRD29B:LUC* construct*)* was induced by 10 µM exogenous ABA. Protoplasts were transfected with DNA of the effector expression cassettes (0 to 3 µg DNA/10^5^ protoplasts) and incubated for 16 h at 25 °C before determination of the reporter activity (RLU) normalized to a cotransfected control (RFU). Data are mean ± SD, n = 3. **b**, GFP fluorescence in nuclei (n = 12) of yeast cells expressing GFP-TWA1 grown at 20 °C and 35 °C. The GFP signal was normalized to background fluorescence. Differences were statistically not significant (two-sided Mann-Whitney U Test, *P* = 0.30). Box plots: centre line, median; box limits, upper (25^th^ percentile) and lower quartils (75^th^ percentile); whiskers, 1.5 times interquartile range. Statistical significance in SI_Extended Fig. 5. Source Data

**Extended Data Fig. 6:**
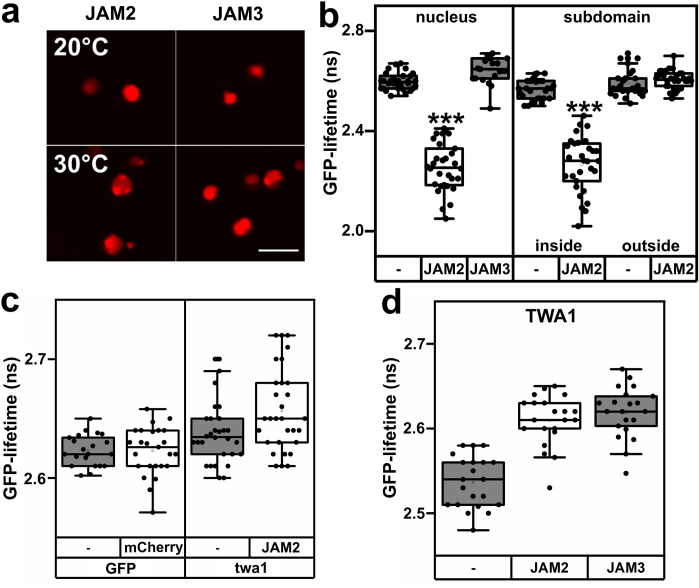
Analysis of JAM2 and JAM3 in yeast. **a**, Fluorescence of mCherry-tagged JAM2 and JAM3 in yeast nuclei at 20 °C (Top) and 30 °C (Bottom). Scale bar 5 µm. **b**, FRET-FLIM analysis of GFP-TWA1 with mCherry-tagged acceptor proteins JAM2, JAM3, or no acceptor (−) at 30 °C in the yeast nucleus. Reduction of TWA1 fluorescence lifetime by JAM2 expression indicates proximity of the proteins which occurred within nuclear subdomains (see **a**). Asterisks indicate a significant difference (JAM2, nucleus, *P* = 0.009; JAM2, inside subdomain, *P* = 0.008; two-sided Mann-Whitney U test, 20-30 cells). **c**, Protein interaction analysis of mCherry-tagged JAM2 with GFP and GFP-twa1 product (twa1). The analysis with non-fused mCherry protein is shown as a control (−). Two-sided Mann-Whitney U-Test, *P* = 0.82 (JAM2, twa1); *P* = 0.68 (mCherry, GFP); n = 20−30. **d**, No detectable interaction of TWA1 with JAM2 at 20 °C in yeast nucleus. FRET-FLIM analysis with GFP-TWA1 (−) and GFP-TWA1 with mCherry-tagged acceptor proteins JAM2 and JAM3 (two-sided Mann-Whitney U-Test, *P* = 0.24 (JAM2, TWA1); *P* = 0.07 (JAM3, TWA1); n = 20−30. Box plots as in SI Extended Data Fig. 5. Statistical significance in SI_Extended Fig. 6. Source Data

The ABA-hypersensitive reporter expression in *twa1* mutants (Fig. 1c and Extended Data Fig. 2) implied that TWA1 has a gene-regulatory role. Transient TWA1 expression in leaf protoplasts resulted in a dose-dependent downregulation of an ABA-responsive reporter (Fig. 2a). The inhibitory response required functional TWA1 and was moderate in WT protoplasts but more conspicuous in protoplasts of *twa1-1* and *twa1-2* plants. The TWA1-mediated inhibition occurred irrespective of exogenous ABA and was temperature dependent (Fig. 2b,c). There was no detectable inhibition by TWA1 at 20 °C, while increasing the incubation temperature from the standard 25 °C to 30 °C increased the repressive effect (Fig. 2b). Homologues of TWA1 are found in monocots and dicots (Extended Data Fig. 3b). The coding sequences of TWA1 homologues from the cold acclimated lyrate rock cress *Arabidopsis lyrata* and white mustard *Sinapis alba*, which naturally occurs in Mediterranean climates, were isolated and *Al*TWA1 and *Sa*TWA1 were expressed in *twa1-2* protoplasts (Fig. 2c). At 30 °C, they inhibited expression of the ABA-responsive reporter, consistent with a conserved function as orthologues. However, the temperature dependence differed with half-maximum inhibitory values (IT_50_) of approximately 20 °C, 26 °C and 30 °C for *Al*TWA1, TWA1 and *Sa*TWA1, respectively. High variability between the *A. thaliana* and *A. lyrata* orthologues occurs in a stretch of 20 amino acids within an amino-terminal region of TWA1 named HVR (Fig. 1d). We reasoned that the HVR might contribute to thermal sensing. Exchanging the HVR for the *Al*TWA1 domain in TWA1 was sufficient to change the thermoresponse of the TWA1(*Al*HVR) chimera to mimic that of *Al*TWA1 (Fig. 2c). The truncated TWA1(ΔN) with deletion of amino acid residues 1–554 was inactive in inhibiting gene expression (Fig. 2c). The findings imply a thermosensory function of TWA1 involving the HVR. The presence of EAR motifs suggested that TWA1 might function as a transcriptional coregulator like the NOVEL INTERACTOR OF JAZ^26^. The search for TWA1-interacting proteins using the yeast-two-hybrid (Y2H) system and indexed open-reading-frame libraries^27^ resulted in the identification of the JAM2 TF (Extended Data Fig. 4). JAM2 is involved in signalling of the phytohormone jasmonate (JA) by antagonizing the JA-promoting action of the TF MYC2, both as a homo- and heterodimer^28,29^. The related TFs JAM1–AIB^30^, JAM3 and MYC2 did not interact with TWA1 in the yeast analysis (Extended Data Fig. 4). The specific JAM2 binding to TWA1 was corroborated in yeast using Förster resonance energy transfer–fluorescence lifetime imaging (FRET–FLIM). FRET analysis of fluorophores requires proximity in the nanometre range (<10 nm) and it reduces the fluorescence lifetime (FL) of the excited donor fluorophore. Changes in the fluorophore distance affect the FRET signal by a power of six^31^. We used the protein fluorophores mono enhanced GFP (GFP) and mCherry with GFP–TWA1 as donor and mCherry-tagged JAM2 or JAM3 as an acceptor. GFP–TWA1 is functional (Extended Data Fig. 5a) and showed a preferential nuclear localization in yeast (Fig. 2d,e). TWA1 abundance was not significantly affected by temperature (Extended Data Fig. 5b); however, temperature affected nuclear TWA1 crowding. At 30 °C and 35 °C, GFP–TWA1 accumulated in nuclear subdomains, which was not observed at 20 °C or for the truncated product of *twa1-1* (twa1 product, Fig. 2d). mCherry-tagged JAM2 and JAM3 revealed a nuclear localization (Extended Data Fig. 6a). TWA1 physically interacted with JAM2 at 30 °C specifically within the nuclear subdomains, whereas JAM3 never detectably did (Extended Data Fig. 6b). JAM2 interaction was not observed with the twa1 product (Extended Data Fig. 6c) and not with TWA1 at 20 °C (Extended Data Fig. 6d) and therefore correlated with TWA1 aggregation in nuclear subdomains. In contrast to GFP–TWA1, GFP–TWA1(*Al*HVR) accumulated in nuclear subdomains already at 22 °C, consistent with a regulatory role of the HVR domain for TWA1 crowding (Fig. 2d). Intramolecular FRET–FLIM analysis of TWA1 in live yeast revealed proximity of the amino and carboxy termini at 17 °C (Fig. 2e). An increase in temperature moved the terminal domains of the sensor apart, as evidenced by the FL increase.

**Extended Data Fig. 7:**
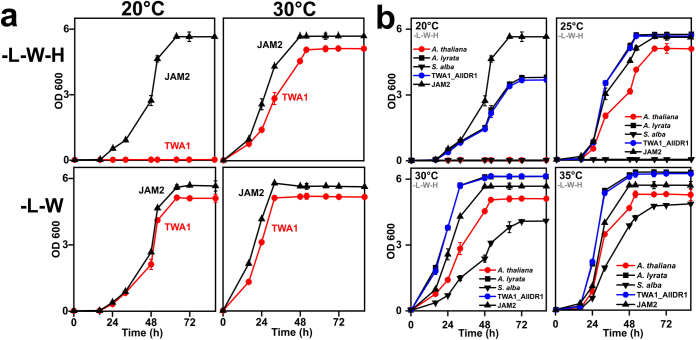
Dependency of JAM2 interaction with TWA1 and TWA1 orthologues on temperature. **a**, Temperature dependence of JAM2 binding to TWA1 and variants. Y2H growth assay for histidine autotrophy. The JAM2-JAM2 pair (JAM2) served as positive control. **b**, Histidine autotrophic yeast growth in the Y2H assay provided by the binding of JAM2 fusion with Gal4-BD to Gal4-AD fusions of JAM2 (JAM2), TWA1, and orthologues from *A. lyrata, S. alba*, and a TWA1 version in which the Arabidopsis HVR has been exchanged for the corresponding domain of *A. lyrata* (TWA1_AlHVR). **a**, **b**, The inoculum was 0.020 optical density (OD) at wavelength 600 nm in growth medium lacking leucine, tryptophan, and histidine (-L,-W,-H), and data are mean ± SD, n = 3 per data point). Source Data

As protein–protein interaction in the Y2H^32^ system provides histidine-autotrophic growth, this read-out enabled examination of the temperature dependence of JAM2 binding to TWA1. At 20 °C, little to no growth occurred with the JAM2–TWA1 pair compared with the dimer-forming JAM2–JAM2 combination under the histidine-selective condition (Extended Data Fig. 7a). Increasing the incubation temperature to 30 °C resulted in histidine-autotrophic growth of JAM2–TWA1-expressing yeast, consistent with the physical interaction of both proteins at permissive temperatures. Thus, yeast growth rates at different temperatures provided a proxy for JAM2–TWA1 interaction, with JAM2 dimerization serving as a reference (Fig. 2f and Extended Data Fig. 7b). In the temperature interval of 20 °C to 25 °C, TWA1 switches from undetectable to close to maximum JAM2 interaction. JAM2 did not bind to the *twa1* product, whereas it interacted with TWA1(ΔN), deleted for the first 554 amino acid residues, at all temperatures (Fig. 2f). In contrast to TWA1, *Al*TWA1 supported yeast growth at 20 °C, whereas *Sa*TWA1 required temperatures of around 30 °C for optimal growth (Fig. 2g and Extended Data Fig. 7b). TWA1(*Al*HVR) and *Al*TWA1 were indistinguishable in this analysis (Fig. 2g), consistent with the results of the plant cell analysis of the thermal characteristics of both sensor proteins (Fig. 2c). The findings reveal a role of the amino-terminal HVR for the temperature-dependent regulation of JAM2 binding to the carboxy-terminal half of TWA1.

## TWA1-mediated transcriptional regulation

**Fig. 3:**
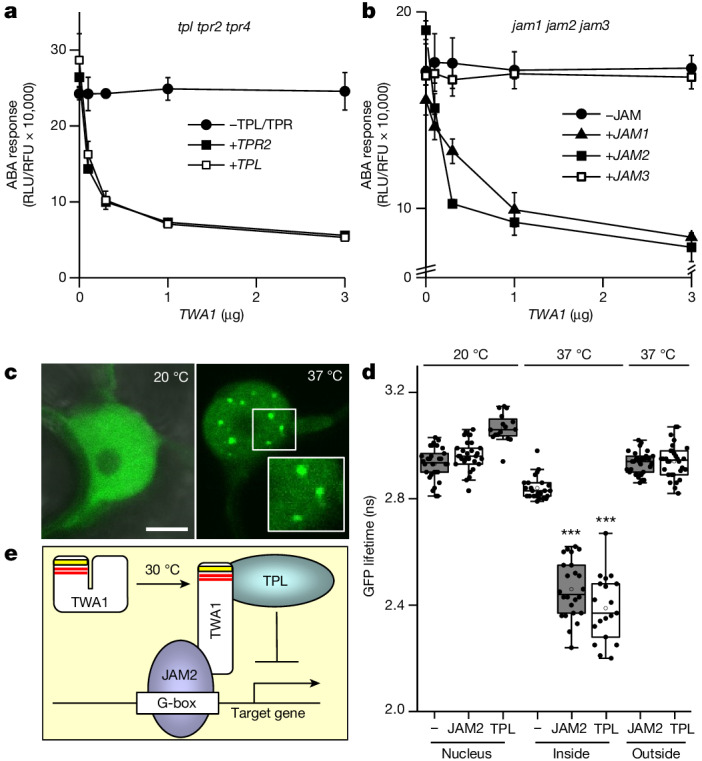
The molecular mechanism of TWA1-mediated transcriptional repression. **a**, TPL and TPR corepressors are required for TWA1-inhibited reporter expression. Protoplasts of *tpl* *tpr2* *tpr4* triple-mutant plants were transfected with *TWA1*, *TPL* and *TPR2* effector DNA. **b**, JAM1 and JAM2 co-expression rescues the TWA1 response in protoplasts of *jam1* *jam2* *jam3* plants. For **a**,**b**, ABA response analysis was performed as in Fig. 2b with 3 µg effector DNA per 10^5^ protoplasts. **c**,**d**, Accumulation and binding of GFP–TWA1 to JAM2 and TPL in nuclear subdomains of *N. benthamiana* epidermal cells. GFP–TWA1 and mCherry-tagged JAM2 or TPL were transiently expressed in leaves exposed to 37 °C for 2 h or kept at 20 °C. **c**, GFP imaging in the nucleus (see the ‘Statistics and reproducibility’ section in the Methods). Scale bar, 5 μm; insets are 1.5-fold magnifications. **d**, FRET–FLIM analysis. Statistical analysis was performed using two-sided Mann–Whitney *U*-tests; ***P = 0.005. *n* = 20–30 cells. Box plots are as described in Fig. 2e. **e**, Simplified model of repressor complex formation. Temperature transitions from 20 °C to 30 °C induce conformational changes in TWA1 that allow JAM2 binding to the carboxy-terminal part of TWA1 and TPL binding to the amino-terminal domain through EAR motifs (red). The HVR (yellow) is integral to thermosensing. JAM2 interacts with G-box-related *cis-*elements as dimers. TPL is tetrameric and targets subunits of the Mediator complex^33^. Statistical significance details are provided as Source Data. Source Data

**Extended Data Fig. 8:**
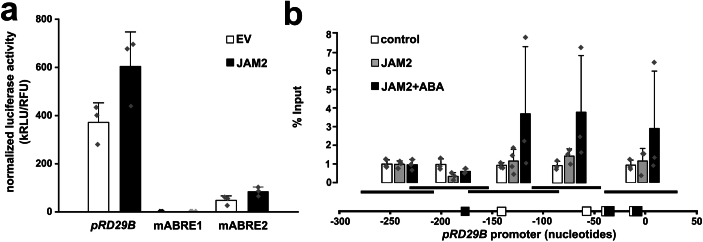
Transactivation and binding of JAM2 to the vicinity of the transcription start site within the RD29B promoter. **a**, Enhanced ABA response by ectopic expression of JAM2. Protoplasts of wild type were transfected with JAM2 effector cassette (3 µg DNA/10^5^ protoplasts) and the promoter *pRD29B* or *pRD29B* with mutated ABA-responsive cis elements (ABRE), mABRE1 or mABRE2, driving luciferase expression. After incubation in the presence of 10 µM ABA for 16 h, the luciferase activity was measured (kRLU/RFU), (mean ± SD, n = 3). **b**, JAM2 interaction with *RD29B* is stimulated in the presence of ABA. Top, ChIP analysis of the promoter region of *RD29B*. Bottom, Scheme of *pRD29B* with the transcriptional start site at position 1. ABREs (motif ACGTG) are signified by filled boxes and their nucleotide distance relative to the start site is given with ABRE1 and ABRE2 closest and next closest to the start site respectively. Potential JAM2 binding sites, that is imperfect G-boxes with one nucleotide deviation from the core CACGTG motif^83^, are indicated with open boxes. Middle, the positions of the amplified DNA fragments are shown. Protoplasts were analysed after transient expression of JAM2 effector (3 µg DNA/10^5^ protoplasts) or not (control) with or without 10 µM ABA (mean ± s.e.m., n = 3-4). Source Data

The EAR domain provides an interaction surface for the co-repressors TPL and TPRs, which suppress transcription and interact with the Mediator complex^24,33^. Y2H analysis revealed binding of TPL/TPRs to TWA1 (Extended Data Fig. 4). The TWA1 inhibitory action on ABA-dependent gene expression required TPL/TPRs in *A. thaliana* protoplasts. TWA1 did not inhibit reporter expression in the triple *tpl* *tpr2* *tpr4* mutant (Fig. 3a). Expression of TPL or TPR2 recovered TWA1-mediated gene regulation in the triple mutant, confirming a function of the co-repressors in the TWA1-mediated downregulation (Fig. 3a). Similarly, protoplasts of the *jam1* *jam2* *jam3* triple mutant were unresponsive to ectopic TWA1 expression unless JAM1 or JAM2 were co-expressed (Fig. 3b). JAMs and MYC2 are basic helix-loop-helix TFs that compete for binding to G-box *cis-*elements and related motifs^28,29^ that include the ABA-responsive regulatory element^34^. Chromatin immunoprecipitation revealed that JAM2 binds to the ABA-responsive *RD29B* promoter close to the transcription start site and this interaction depended on both ABA and an ABA-responsive regulatory element (Extended Data Fig. 8a,b).

The physical interaction of TWA1 with TPL and JAM2 was confirmed using FRET–FLIM in *Nicotiana benthamiana*. Transient expression of GFP-tagged TWA1 in epidermal cells showed a nuclear localization of TWA1 that accumulated in subdomains (Fig. 3c), similar to the yeast analysis (Fig. 2d). FRET–FLIM supported binding of TPL to TWA1 and corroborated the TWA1–JAM2 complex formation at 37 °C (Fig. 3d). Both interactions occurred specifically in these nuclear subdomains but not at 20 °C (Fig. 3d). The data support the model in which elevated temperatures induce intramolecular rearrangements of TWA1 that allow interaction with JAM2 and TPL for assembly of the transcriptional repressor complex (Fig. 3e) in nuclear subdomains.

## Thermotolerance conferred by TWA1

**Fig. 4:**
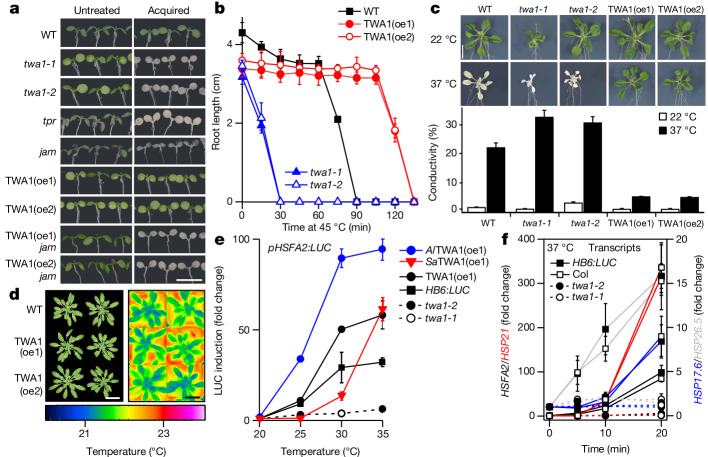
Thermotolerance and heat shock transcripts are controlled by TWA1. **a**, Acquired thermotolerance depends on TPL/TPR-type corepressors and on JAM TFs with loss of thermotolerance conferred by TWA1 expression (TWA1(oe) lines) in *jam1* *jam2* *jam3* (*jam*) plants. TWA1 was expressed under the viral 35S promoter in *twa1-2* plants. *tpr* signifies the *tpl* *tpr2* *tpr4* genotype. Photographs were taken 1 day after heat stress (150 min, 45 °C) or without heat stress. Scale bar, 1 cm. **b**, The basal thermotolerance of 5-day-old seedlings. Root growth analysis as in Fig. 1b but without priming. **c**, Ion leakage of 3-week-old plantlets that were exposed to 37 °C for 24 h or kept at 22 °C. Ion leakage was expressed relative to maximum leakage by boiling. Data are mean ± s.d. *n* = 12 from three repetitions. Scale bar, 1 cm. **d**, Improved thermotolerance of TWA1(oe) lines without trade-offs in growth or changes in apparent photosynthetic parameters (Extended Data Fig. 9d,e). Photograph of plants grown for 38 days under short-day conditions at 22 °C and thermal images in false colours. Scale bars, 3 cm. **e**, Temperature- and TWA1-dependent induction of the HSFA2 promoter driving LUC expression in leaf protoplasts of different lines. Analysis was performed as in Fig. 3a. Expression of *Al*TWA1 and *Sa*TWA1 in *twa1-2* plants. Data are mean ± s.d. *n* = 3 per datapoint. **f**, Transcript changes in 5-day-old WT and *twa1* seedlings grown at 20 °C and exposed to 37 °C for up to 20 min. The transcript level at *t* = 0 was set to 1. Data are mean ± s.d. *n* = 3, 10 seedlings per *n*. Statistical analysis details for **b**,**c**,**e** are provided as Source Data. Source Data

**Extended Data Fig. 9:**
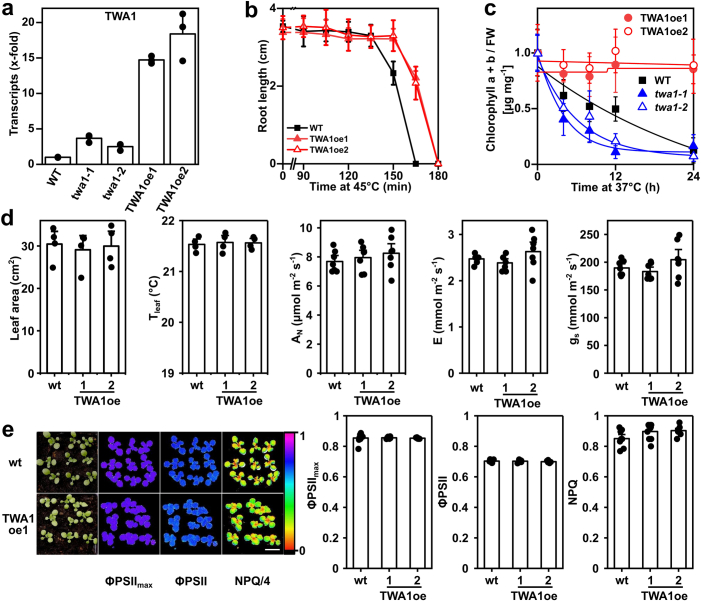
Ectopic expression of TWA1 enhances thermotolerance but does not affect growth and photosynthesis. **a**, *TWA1* transcript levels in two representative TWA1-overexpressing (TWA1oe) lines in comparison to wild type and the *twa1* mutants. 5-d old seedlings were analysed for TWA1 transcript abundance by RT-qPCR with UBC9, UBI10 and TIP41L transcripts as reference. Ectopic *TWA1* expression was under the control of the viral 35 S promoter in *twa1-2* mutant background. The TWA1 transcript level in wild type (*pHB6:LUC*) was set to 1 and fold change of abundance in mutants and TWA1oe lines is presented. Data are mean ± SD, n = 3, ten seedlings per n. **b**, Enhanced acquired thermotolerance of TWA1oe lines. Inhibition of root growth in TWA1oe lines (red) compared to WT in response to various 45 °C exposure times (mean ± s.d., n = 6). **c**, Basal thermotolerance is improved in TWA1oe lines and impaired in *twa1-1* and *twa1-2*. Chlorophyll content per fresh weight of 5d-old seedlings one day after exposure to 37 °C as indicated (mean ± s.d., n = 15 from three repetitions). The chlorophyll content at t = 0 was set to 1. **d, e**, Improved thermotolerance of TWA1oe lines without tradeoffs in growth and changes in apparent photosynthetic parameters. Comparison of total leaf area (leaf area; n = 4), leaf temperature (T_leaf_; n = 4), net photosynthesis (A_n_; n = 7), transpiration (E; n = 7), and stomatal conductance (g_s_; n = 7) of plants grown for 38 days at short-day conditions with 22 °C daytime temperature and 150 µmol m^−2^ s^−1^ photosynthetic active radiation (mean ± s.e.m.). **e**, 20-day-old plantlets analysed for maximum quantum efficiency of photosystem II (FPSII_max_), quantum efficiency of photosystem II (FPSII), and non-photochemical quenching (NPQ). No statistically significant differences were found (n = 9; *P* > 0.05, one-way ANOVA). Left, Values are expressed in false colours. Right, mean ± s.e.m. Statistical analysis for **b-e** is provided in SI Extended Data Fig. 9. Source Data

In agreement with the functional interactions of TWA1 with TPL/TPR and JAM proteins, seedlings of the *jam1* *jam2* *jam3* and *tpl* *tpr2* *tpr4* triple mutants showed a compromised acquired thermotolerance like *twa1-1* and *twa1-2* plants (Fig. 4a). Ectopic *TWA1* expression under the viral 35S promoter resulted in 15- and 18-fold increased *TWA1* transcript levels in the *twa1-2* mutant compared with in the WT in two representative TWA1-overexpression (TWA1(oe)) lines (Extended Data Fig. 9a), which exhibited higher acquired (Fig. 4a and Extended Data Fig. 9b) and basal (Fig. 4b) thermotolerance. The thermotolerance conferred by TWA1 expression depended on JAMs, as shown by the thermosensitivity of TWA1(oe) lines in the *jam1* *jam2* *jam3* genotype (Fig. 4a). *A. thaliana* seedlings that were exposed to 37 °C responded with chlorophyll loss that was enhanced in *twa1* mutants and ameliorated by ectopic TWA1 expression compared with the WT (Extended Data Fig. 9c). The improved basal thermotolerance of the TWA1(oe) lines was also evident in the reduced ion leakage of 3-week-old plantlets that were exposed for 24 h to 37 °C (Fig. 4c). Notably, the TWA1(oe) lines cultivated at ambient conditions were indistinguishable from the WT in growth, photosynthesis and gas-exchange parameters (Fig. 4d and Extended Data Fig. 9d,e).

**Extended Data Fig. 10:**
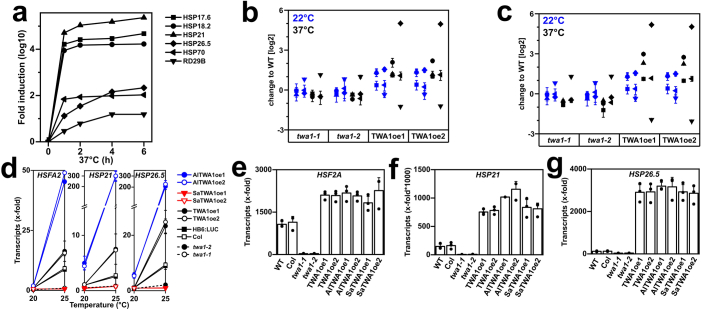
Transcript levels in seedlings expressing TWA1 or TWA1 orthologs in comparison to wild type and twa1 mutants. **a**, Left, Changes in transcript abundance for HSPs and ABA-induced RD29B in wild type of 5 days-old seedlings exposed to a shift from 22 °C to 37 °C for up to 6 h. Transcripts were quantified by realtime PCR after 4 h recovery at 22 °C. The transcript level in WT (*pHB6:LUC*) without heat treatment was set to 1. **b**, Transcript changes in TWA1-deficient and TWAoe lines compared to WT as in **a**, with 37 °C exposure for 4 h (treatment and symbols as in **a**. Transcripts were analysed by RT-PCR). **c**, Analysis as in **b**, however, with a recovery time of 0.5 h. **d**, Transcript changes of *HSFA2*, *HSP21*, and *HSP26.5* affected by TWA1 and orthologs in 5-d old seedlings from ten different lines grown at 20 °C and exposed for 1 h to 25 °C, or kept at 20 °C, with 0.5 h recovery period at 20 °C prior to RT-qPCR analysis. **e-g**, Transcript abundance in *A*. *thaliana* seedlings grown at 20 °C and exposed to 35 °C for 1 h after 30 min recovery at 20 °C that expressed TWA1, the orthologues from *A. lyrata* AlTWA1 and *Sinapis alba* SaTWA1 under the control of the viral 35 S promoter in the *twa1-2* background. The analysis of two representative lines is shown in comparison to WT lines (*pHB6:LUC* and Col-0) and both *twa1* mutants for **e**, *HSFA2*, **f**, *HSP21* and **g**, *HSP26.5* transcript levels. The transcript abundance of WT *pHB6:LUC* line at 20 °C is set to 1. **a-g**, Data are mean ± s.d., n = 3, ten seedlings per n. Statistical significance in SI_Extended Fig. 10. Source Data

The enhanced thermotolerance by TWA1 expression might be caused by a preinduced HSR due to constitutive upregulation of *HSP* transcripts^35–41^. We examined the possibility. In WT seedlings, a temperature shift to priming periods at 37 °C induced transcripts for small HSPs and the major ATP-dependent chaperone HSP70 as part of the HSR (Extended Data Fig. 10a). At ambient temperatures, the levels of these transcripts were similar to the WT in TWA1-deficient and TWA1(oe) lines, therefore not supporting preinduction of HSR transcripts in the TWA1(oe) lines (Extended Data Fig. 10b,c). A 4 h priming phase increased *HSP* transcript levels in the TWA1(oe) lines up to 30-fold higher for *HSP26.5* compared with the WT, while  the increases were marginally reduced in both *twa1* mutants (Extended Data Fig. 10b,c). Given the IT_50_ value of TWA1 around 26 °C, the induction of early HSR transcripts was monitored at lower temperatures. First, we used the activation of the *HSFA2* promoter as a readout. The *HSFA2* promoter is directly targeted by the master HSF1-class TFs, HSFA1s in plants, and the HSFA2 induction is HSFA1 dependent and sufficient for establishing heat tolerance^36,37^. In WT protoplasts, a shift from 20 °C to 25 °C increased *HSFA2-*promoter-driven LUC expression with optimal values at 30 °C and 35 °C (Fig. 4e).

The induction did not occur in *twa1* protoplasts and was enhanced in TWA1(oe) cells (Fig. 4e). A thermal response shift to lower and higher temperatures occurred in protoplasts from *Al*TWA1(oe1) and *Sa*TWA1(oe1) plants expressing the lyrate and mustard orthologue in the *twa1-2* genotype compared with protoplasts of the WT and TWA1(oe) plants (Fig. 4e). The TWA1 dependence of the response was corroborated by transcript analysis of seedlings that were exposed to a 1 h temperature shift from 20 °C to 25 °C (Extended Data Fig. 10d). Compared with the WT, the transcript increase for *HSFA2*, *HSP21* and *HSP26.5* was supressed in *twa1-1*, *twa1-2* and *Sa*TWA1(oe) seedlings, while it was enhanced in TWA1(oe) and *Al*TWA1-expressing seedlings (Extended Data Fig. 10d). At 35 °C, the transcript levels were comparable in *Sa*TWA1(oe), TWA1(oe) and *Al*TWA1(oe) seedlings and consistently higher than in the WT (Extended Data Fig. 10e–g). To further elucidate the role of TWA1, WT and TWA1-deficient *A. thaliana* seedlings were exposed to 5 up to 20 minutes of heat stress (Fig. 4f). The transcript levels of *HSFA2*, *HSP17.6*, *HSP21* and *HSP26.5* were clearly induced in both WT lines but did not change in both *twa1* mutants, in agreement with a positive regulatory function of the thermosensor for the upregulation of HSR transcripts.

In summary, our study reveals a thermosensor function of TWA1 that is required for heat tolerance and timely induction of *HSFA2* and *HSP* transcripts in *A. thaliana*. The HSR is triggered by activation of key HSF1-type TFs. In *A. thaliana*, three related HSFA1s redundantly initiate the HSR^38^. The combined inactivity of two of those master TFs still allows HSFA2 induction and thermotolerance to heat-induced ion leakage^39,41^. Both responses are impaired in plants with TWA1 deficiency, supporting an action of the thermosensor upstream of HSFA1s or, alternatively, in parallel. Ectopic expression of constitutively active HSFA1 TFs^39,40^ and of TWA1 provide improved thermotolerance. Constitutive heat tolerance by HSFA1 is associated with preinduced HSR and growth penalty^39^, traits that are also conferred by ectopic HSFA2 and HSFA3 expression^35,37^. However, neither adverse physiological effect was observed in TWA1-mediated heat tolerance. Moreover, the induction of HSR transcripts within minutes required a functional TWA1. The findings point to an inducible protective function of TWA1 in early signalling in response to temperature rise.

Thermal activation of TWA1 involves the HVR and temperature-induced domain rearrangements that control the binding of JAM2 and repressor proteins. The role of TWA1 bears parallels to the thermosensor EARLY FLOWERING 3 (ELF3)^6^. ELF3 is a component of the evening complex, which functions as a transcriptional co-repressor in the control of the circadian clock and flowering time in *A. thaliana*. Thermally, TWA1 acts in the opposite manner to ELF3 by repressing gene expression at elevated temperatures, and it responds more sensitively to temperature changes. The TWA1 response has an apparent *Q*_10_ value of around 150, that is, a 10 °C difference can induce a 150-fold change in activity as deduced from growth rates in Y2H analyses (Methods). This responsiveness of TWA1 is in the range of the most thermosensitive proteins known, including members of the TRPA1 Ca^2+^channel of snake^42^ and mosquitos^43^ that are used for thermal prey localization. In eukaryotes, heat stress induces cytosolic and organellar stress granules pioneered in plants^44^ and the formation of nuclear subdomains that are reversible aggregates of proteins and RNA for cellular adaptation to thermal challenges^8,45–47^. Liquid–liquid phase separation (LLPS) has a prominent role in the formation of multilayered nuclear subdomains as sites of active gene expression^48^. After thermal activation, TWA1 accumulated in nuclear subdomains that are reminiscent of LLPS. These subdomains are the sites for TWA1 interaction with JAM2 and TPL, indicating the formation of active repressor complexes specifically in the presumed LLPS. Proteins with intrinsically disordered regions have a propensity for LLPS^49^ and include plant proteins with a prion-like domain^50,51^. Under heat stress, the intrinsically disordered region of the yeast HSF1 promotes condensate formation and multiple chromatin interactions for transcription within a single LLPS^52^. The temperature-sensing mechanism leading to HSF1 activation in yeast and mammals is still not clarified, while there is mounting evidence for the chaperone HSF1 titration model^8^.

TWA1-type sensors might be unique to plants. TWA1 sensed sub-heat temperature elevations between 20 °C and 30 °C and was required for the induction of early HSR transcripts. However, after a 4 h priming phase, *HSP* transcript levels were comparable between the WT and *twa1* mutants, implying additional modes of transcript upregulation. In plants, heat-triggered increases in hydrogen peroxide^53,54^ and nitrogen oxide^55^ activate HSFA1s and allow for redox signal integration into the HSR. TWA1 possibly provides another node of signal integration. Plants frequently experience combined stress with the challenge to integrate conflicting single stress responses^56^. For example, the ABA-mediated proline increase under water deficit provides protective osmolytes, but high proline levels are toxic under heat^57^. TWA1 probably orchestrates the acclimation responses by integrating temperature with ABA and JA signalling^58^ that relay abiotic and biotic cues, respectively. In the future, using TWA1-type temperature sensors with different thermal characteristics through breeding and biotechnology will enable the adjustment of the acclimation responses of crops to a warming climate^59^. Moreover, TWA1 and its orthologues provide prototypes for engineering temperature switches in the emerging field of thermogenetics^60^.

## Methods

### Plant materials and growth conditions

Plants of *A. thaliana* Heynh. ecotype Col and La-er were grown in pots on a perlite/soil mixture at 22 °C under long-day conditions with 16 h light (150 µE m^−2^ s^−1^) unless otherwise stated. The plants were used for stable transformation, protoplast preparation and DNA extraction. *A. thaliana* seedlings were grown on agar plates with half-strength Murashige and Skoog agar medium for physiological assays as previously described^61^. For thermotolerance tests, growth conditions were as reported previously^14^. T-DNA knockout lines of the GABI-Kat collection^62^, including GK-476H03 (*At5g13590*; *twa1-2*) and SALK collection^63^ including SALK_143411 (*At2g33540*; designated *cpl3-10*) were obtained from The European *Arabidopsis* Stock Centre.

The *JAM2/bHLH013* (*At1g01260*) gene was inactivated in the *pHB6:LUC* line using the CRISPR–Cas9 system^64,65^ and vector pDGE63, (Addgene plasmid, 79445). Two independent *jam2* isolates contained a frameshift, which resulted in early termination of translation after amino acid 12 and we designated them as *jam2-2*. DNA-sequencing analysis revealed the absence of off-target mutations in the closely related genes *JAM1/bHLH017* (*At2g46510*) and *JAM3/bHLH003* (*At4g16430*). The triple mutant *jam1-2* *jam2-2* *jam3-2* (*jam*) was generated by crossing *jam2-2*, *jam1-2* (GK_285E09) and *jam3-2* (GK_301G05)^28^ plants and selecting for the homozygous *jam* mutant in the offspring. Similarly, the *tpl-8* *tpr2-2* *tpr4-2* triple mutant (*tpr*) was obtained by crossing *tpl-8* (SALK_036566) with SALK_112730 (*tpr2-2*) and SALK_002209 (designated *tpr4-2*) plants. The generation of RCAR1- and RCAR6-overexpressing lines has been reported previously^61^. Quadruple RCAR-knockout lines were obtained by combining the multiple PYR/PYL knockout line *pyr1* *pyl1* *pyl2* *pyl4* *pyl5* *pyl8*^66^, the *rcar9* mutant^67^ and lines SALK_083621 (*rcar1*) and GK-012D02 (*rcar13*). The triple mutants *abi1-2* *abi2-2 hab1-1* (*pp2c_a*) and *abi1-2* *hab1-1* *pp2ca-1* (*pp2c_b*)^19^ were a gift from P. L. Rodriguez together with the multiple PYR/PYL knockout. Mutants were crossed to the ABA reporter lines *pRD29B:LUC* and *pHB6:LUC*^23^. Lines homozygous for reporter constructs and for mutant alleles were used throughout the experiments.

### Mutant isolation and ABA exposure

Mutants with ABA-hypersensitive reporter activation were recovered from a screen of the maternal second generation (M_2_) of ethyl methanesulfonate-mutagenized *A. thaliana* seeds of the ABA reporter line *pHB6:LUC*^23^ in the Col-0 background. In brief, the M_2_ seedlings were grown on solidified half-strength MS medium for 5 days before transfer to medium supplemented with 3 µM (+)-*cis-trans*-ABA (Chemos; www.chemos-group.com) or 0.3 M mannitol for 24 h followed by life luciferase imaging with the intensity of light emission from plant organs of interest being measured using Simple PCI 6.6 software (https://hcimage.com/)^23^. Among the mutants recovered, certain mutants were hypersensitive to exogenously applied ABA, while mutants with an unaltered response to ABA were considered to carry lesions in drought stress signalling upstream of the canonical ABA signalling pathway. About 115,000 seedlings were screened and 109 putatively hypersensitive mutants were selected. Candidates showing a hypersensitive reporter response were propagated and the progeny was re-examined. Finally, 24 mutants were confirmed, among which 16 were found to be ABA-hypersensitive and 8 were found to be affected in drought stress signalling upstream of ABA. We selected mutants with a robust phenotype for map-based cloning in combination with next-generation sequencing and identified *twa1-1* together with mutants allelic to *cpl1* and *cpl3*. The *twa1-1* mutant was backcrossed to the *pHB6:LUC* reporter line four times.

### Gene identification

The *TWA1* locus has been identified by bulked segregant analysis^68^. In brief, approximately 50 homozygous mutant seedlings were pooled and processed for next-generation DNA-sequencing analysis to identify single-nucleotide polymorphisms (SNPs) compared with the reference genome Col-0. The analysis confined the location of the target gene within a 180 kb genomic fragment on chromosome 5. In this fragment, four SNPs were found within genes, two of them were synonymous. The non-synonymous mutations generated a premature TGA stop codon in *At5g13590* at nucleotide 744 of the coding sequence and the other caused a conservative amino acid exchange in *At5g13930*. The identity of *TWA1* (*At5g13590*) was confirmed by complementation of the ABA-hypersensitive phenotype by gene transfer of a 7 kb genomic fragment encompassing 1.1 kb of the promoter region, the structural gene and the terminator (a list of the primers and restriction sites used for cloning is shown in Supplementary Table 1).

### Effector constructs and analysis of gene expression

Plant RNA extraction, cDNA synthesis and construction of plasmids for effector expression were performed as described previously^61^. In brief, cDNA was generated from mRNA isolated from leaves of *A. thaliana* Col-0, *A. lyrata* and *S. alba*. Total RNA was purified from leaves using the analytik jena-innuPREP Plant RNA Kit (Analytik Jena) and cDNA was synthesized using the RevertAid First Strand cDNA Synthesis Kit (Thermo Fisher Scientific). The coding sequences of effectors were integrated into a modified Bluescript vector with an expression cassette consisting of the 35S promoter, followed by the coding sequence and the NOS terminator^61^. A list of the primers and restriction sites used for cloning is provided in Supplementary Table 1. The 363 bp AvrII–HindIII DNA fragment of the Al*TWA1* gene encompassing the HVR was exchanged with the AvrII–HindIII fragment of *TWA1* to yield *TWA1(AlHVR)*. Constructs used for FRET–FLIM analysis were cloned using the Golden-Gate system^69^. *TWA1*, *twa1-1*, *JAM2* and *JAM3* cDNAs were cloned into the level I vector (LI_BpiI) and subsequently into LII expression vectors (*JAM2* and *JAM3*: LII_3-4_CEN_LEU with promoter *pTDH3* and terminator *tDH1*, plus an N-terminal mCherry tag; *TWA1*and *twa1*: LII_1-2_CEN_LEU with *pTDH3* and *tDH1*, plus an N-terminal GFP tag). For intramolecular FRET, *TWA1* was cloned into LII_3-4_CEN_LEU with *pTDH3* and *tDH1*, including an N-terminal mCherry tag and a C-terminal GFP (GFP used in all constructs was mono enhanced GFP) tag linked to TWA1 through a glycine–serine linker (mCherry: GGGGSGGGGSGGGGSG; GFP: GGGGGSGGGGSGGGGS). As a control, both fluorophores were fused with the same glycine–serine linkers and expressed using the LII_3-4_LEU expression cassette. The GFP-TWA1(*Al*HVR) construct was cloned into pGREG574^70^ using the *TWA1*(*AlHVR*) cassette (see above) and SalI. The reporter construct *pHSFA2:LUC* was generated by replacing the *RD29B* promoter in *pRD29B:LUC* with an amplified 2 kb fragment of the *HSFA2* promoter region as described previously^54^. The correctness of all constructs was verified by DNA-sequencing analysis. Quantitative gene expression was monitored with BRYT Green Dye-based qPCR (GoTaq qPCR Master Mix kit, Promega) using the LightCycler 480 instrument (Roche) and gene-specific primers. *TIP41L*, *UBC9* and *UBI10* were used for normalization. A list of the primers used for quantitative PCR with reverse transcription is shown in Supplementary Table 2.

### Protein sequence alignments

Homologues of *TWA1* were identified through BLASTp searches against genomes on NCBI (https://blast.ncbi.nlm.nih.gov/Blast.cgi?PAGE=Proteins). Amino acid sequences of TWA1 and representative proteins were aligned with the NCBI COBALT alignment tool^71^.

### Prediction of intrinsically disordered proteins

Prediction of intrinsically disordered regions was performed using metapredict (https://metapredict.net/)^72^.

### Protoplast assays and transgenic plants

Transient expression analysis in *A. thaliana* protoplasts, and expression cassettes for *pRDB29:LUC*, *p35S:GUS* and effectors have been described previously^73^. Primers for DNA amplification and restriction sites used for generation of effector constructs are listed (Supplementary Table 1). Protoplasts were isolated from leaves of Columbia WT accession (Col-0) or from mutant lines of the Col-0 background. In brief, 10^5^ protoplasts were transfected with DNA of different expression cassettes including the ABA-responsive *pRD29B:LUC* reporter (5 µg), the *p35S:GUS* control reporter (3 µg) for expression normalization and the indicated amounts of various effector plasmids and incubated at 25 °C, unless otherwise stated, for 16 h before assessment of luciferase and glucuronidase activity. Ectopic expression of *TWA1* and *twa1* (mutant allele of *twa1-1*), Al*TWA1* and Sa*TWA1* in *A. thaliana* plants was under the 35S promoter in the *twa1-2* background. The cDNA expression cassettes of the TWA1 variants and orthologues were inserted as an AscI DNA fragment into the pGreenII 0179 vector^74^ modified with an AscI cloning site in the T-DNA region. Transgenic plants were generated by *Agrobacterium tumefaciens*-mediated gene transfer as described previously^75^.

### Thermotolerance evaluation

Thermotolerance was analysed as reported previously^14^. In brief, 5-day-old light-grown seedlings (22 °C) were exposed to heat stress at 45 °C for 90 min to 180 min with 15 min intervals for assessment of basal thermotolerance. For analysis of acquired thermotolerance, seedlings had a preceding 90 min acclimation period at 38 °C followed by a 120 min recovery phase (22 °C) before heat stress at 45 °C. For root growth assays, seedlings were allowed to recover for 5 days under continuous light (22 °C) and the root extension after heat stress was determined.

### Determination of chlorophyll content and of electrolyte leakage

For chlorophyll analysis, the seedlings were frozen in liquid nitrogen after their fresh weight had been determined, homogenized using the TissueLyser II (Qiagen) and extracted with methanol for determination of chlorophyll as reported previously^76^. Absorption of cleared extracts was recorded at 665 nm and 652 nm. In the electrolyte-leakage assay, leaves of 3-week-old *A. thaliana* plants were placed into Petri dishes containing 25 ml water and were incubated at 37 °C or 22 °C in the light (65 µmol m^−2^ s^−1^) for 24 h. Thereafter, single leaves were immersed in 1 ml of double-distilled water in Eppendorf tubes and incubated for 30 min with gentle shaking^9^. Conductivity was measured using a Conductivity Meter (Seven Easy Mettler Toledo, InLab 752-6 mm conductivity sensor). Electrolyte leakage was normalized to the conductivity after heating the samples to 99 °C for 10 min.

### Chromatin immunoprecipitation experiments

GFP–JAM2 was expressed in *A. thaliana* Col-0 protoplasts that were incubated for 16 h in the absence or presence of ABA (10 µM). Protoplasts transfected with an empty vector served as the control. Protoplasts were collected by centrifugation (500*g*, 2 min) and the supernatant was discarded. Subsequent steps (nucleus isolation, shearing of chromatin, preclearing, immunoprecipitation, reverse cross-linking and DNA purification) were performed as described previously^77^. Anti-GFP antibodies were obtained from Santa Cruz Biotechnology (1:2,000 dilution was used for antibodies) and ChromoTek GFP-Trap Agarose was obtained from Proteintech. The enrichment of different DNA fragments encompassing the *RD29B* promoter in the antibody containing fraction (as compared to the control without antibody) was quantified using quantitative PCR from immunoprecipitated samples.

### Photosynthetic parameters and thermoimaging

Gas-exchange measurements and thermoimaging were performed as described previously^18^. For quantifying net photosynthesis (*A*_n_), transpiration (*E*) and stomatal conductance (*g*_s_) of the whole rosette, the GFS-3000 gas-exchange system was equipped with custom-built whole plant cuvettes (Heinz Walz). The analyses were conducted at 150 µmol m^−2^ s^−1^ PAR, 400 µmol mol^−1^ external CO_2_ and a water vapour deficit of 1.3 ± 0.1 kPa using the software of the instrument supplier. PAM imaging was performed by using a MAXI version of IMAGING PAM (Heinz Walz). The operation of the PAM imaging system was performed according to the manufacturer’s instructions. In brief, plants were dark-adapted for 30 min and then subjected to a saturating light pulse, and the maximum quantum efficiency of photosystem II (ϕPSII_max_) was calculated from basic (*F*_0_) and maximum level of fluorescence (*F*_m_). Actinic light was then applied (150 µmol m^−2^ s^−1^ PAR) and, after 1 h of illumination, a saturating light pulse was triggered to determine transient fluorescence (*F*_t_) and maximal fluorescence (*F*_m′_). The corresponding quantum efficiency of photosystem II (ϕPSII) was calculated as (*F*_m′_ − *F*_t_)/*F*_m′_. The non-photochemical quenching (NPQ) was determined as the ratio of (*F*_m_ − *F*_m′_)/*F*_m′_. The images of NPQ are presented using the standard false colour code, with rescaled values (original values divided by 4) ranging from 0 to 1.

### Confocal microscopy

Confocal microscopy and FRET–FLIM analyses were performed as described previously^78^. In brief, leaves of 5-week-old tobacco (*N. benthamiana*) were infiltrated with a suspension of *A. tumefaciens* GV3101 (MP90) for expression of the viral p19 protein and *A. thaliana* proteins. The bacteria contained binary level II plasmids^79^ for the expression of *GFP-TWA1/twa1*, and mCherry fusions with *JAM2*, *JAM3* and *TPL* under the control of the viral 35S promoter in plant leaves. Infiltrated plants were incubated for 2 days at 20 °C and exposed for 2 h to 37 °C or 20 °C. For analysis of yeast, freshly transformed yeast cells of strain AH109 (MATa, obtained from J. Uhrig, Cologne) were cultivated overnight in 1 ml synthetic dextrose medium (SD) at different temperatures as indicated before confocal analysis. Confocal analysis was conducted using the Olympus FluoView 3000 inverse laser-scanning confocal microscope with the UPLSAPO 60XW 60×/NA 1.2/WD 0.28 water-immersion objective (Olympus). For imaging of the GFP and mCherry fluorophore, tissue samples were excited at 488 and 561 nm, respectively. Specific GFP fluorescence in the nucleus was calculated by subtracting the background fluorescence^80^. For FRET–FLIM data acquisition, the PicoQuant advanced FCS/FRET–FLIM/rapidFLIM upgrade kit (PicoQuant) was used. GFP was excited at 485 nm with a pulsed laser (pulse rate, 40 MHz; laser driver, PDL 828 SEPIA II; laser, LDH-D-C-485, PicoQuant), and fluorescence emission was collected using the Hybrid Photomultiplier Detector Assembly 40 (PicoQuant) and processed by the TimeHarp 260 PICO Time-Correlated Single Photon Counting module (resolution, 25 ps; PicoQuant). At least 250 photons per pixel were recorded for each analysed sample. Data were fitted to a bi-exponential decay function and convoluted using SymPhoTime 64 software (PicoQuant).

### Yeast constructs and growth analyses

Yeast growth and Y2H assays were performed as reported previously^67^ unless otherwise stated using pGAD424 (GenBank: U07647) and pBRIDGE vectors for expression of *A. thaliana* proteins (Clontech). A screen for proteins interacting with TWA1 was performed with *A. thaliana* Y2H libraries^27,81^. Coding sequences of *TWA1 *and homologues were expressed as fusions with the GAL4-activation domain (AD), while JAM, MYC2, TPL and TPRs were fused to the GAL4-DNA binding domain. For analysis of yeast growth in liquid culture, precultures of three independently transformed yeast colonies per construct were used for inoculation of 1.5 ml SD medium containing 2% glucose supplemented with 20 mg l^−1^ uracil, 20 mg l^−1^ methionine and, for selection of plasmids and protein interaction, supplemented with 20 mg l^−1^ histidine, 60 mg l^−1^ leucine and 50 mg l^−1^ tryptophan as indicated. After growth overnight in a gyratory shaker at 200 rpm and 30 °C, 13.5 ml supplemented SD was added, and the suspension culture was further cultivated until the beginning of the exponential growth phase was reached at optical densities at 600 nm (OD_600_) of between 0.6 and 0.8. Subsequently, cells were sedimented (1,500*g*, 5 min), resuspend in selective SD and used to inoculate 20 ml fresh SD to a final OD_600_ of 0.020 for monitoring growth at different temperatures (200 rpm). The apparent growth rate *µ* was calculated in the first 24 h of culturing using the formula *µ* = (ln[OD_24 h_/OD_0 h_])/24 h. The growth rate in yeast was used to approximate the *Q*_10_ temperature coefficient of the TWA1 response as *Q*_10_ = [*µ*_T2_/*µ*_T1_]^10/(T2 − T1)^ where *T* is temperature in Celsius and *µ*_T1_ and *µ*_T2_ are the rates at T1 and T2, respectively. The *Q*_10_ value of ~150 was estimated using the measured rates of 0.138 and 0.011 h^−1^ at 25 °C and 20 °C, respectively.

### Statistics and reproducibility

Statistics were derived from data of biological replicates excluding technical replicates. The sample size was calculated using the G*Power v.3.1.9.7 software^82^ for unpaired two-sided *t*-tests, with *n* = 3 for protoplast and yeast experiments with a presumed effect size of 5, *α* and *β* of 0.05, and power (1 − *β*) = 0.95. The number of biological replicates was *n* = 6 for single datapoints in the seedling growth assay with a presumed effect size of 2.5, *α* and *β* of 0.05, and power (1 − *β*) = 0.95. Each experiment was repeated at least twice with similar results. Representative confocal images are shown in Figs. 2d and  3c. In yeast, approximately 85% of fluorescent cells showed the GFP–TWA1 subnuclear crowding at 30 °C, never observed with the twa1 product (Fig. 2d, *n* > 100). In tobacco epidermal cells, the nuclear subdomain accumulation of GFP–TWA1 was 75% at 37 °C (Fig. 3c, *n* > 50). The nuclear TWA1 crowding was consistent in independent experiments. Data were analysed using Mann–Whitney U-tests unless otherwise stated (https://www.socscistatistics.com/tests/mannwhitney and https://www.statskingdom.com/170median_mann_whitney.html, to calculate exact *P* values of <0.0001). One-way analysis of variance was performed using SPSS v.16.0 software for Windows. Student’s *t*-tests were performed using Excel 2016. Box plots were drawn using OriginPro 2020. Details on the statistical analyses are found in the Source Data.

### Reporting summary

Further information on research design is available in the Nature Portfolio Reporting Summary linked to this article.

## Online content

Any methods, additional references, Nature Portfolio reporting summaries, source data, extended data, supplementary information, acknowledgements, peer review information; details of author contributions and competing interests; and statements of data and code availability are available at 10.1038/s41586-024-07424-x.

### Supplementary information

Supplementary Tables 1 and 2

Reporting Summary

### Source data

Source Data Fig. 1

Source Data Fig. 2

Source Data Fig. 3

Source Data Fig. 4

Source Data Extended Data Fig. 1

Source Data Extended Data Fig. 2

Source Data Extended Data Fig. 5

Source Data Extended Data Fig. 6

Source Data Extended Data Fig. 7

Source Data Extended Data Fig. 8

Source Data Extended Data Fig. 9

Source Data Extended Data Fig. 10

## Data Availability

All data supporting the findings of this study are available within the Article. Data on TWA1 protein and transcript abundance in *A. thaliana* organs are from the ATHENA proteomics database (https://athena.proteomics.wzw.tum.de). Source data are provided with this paper.
