# Supplementary material for: The temperature sensor TWA1 is required for thermotolerance in Arabidopsis
Source: Nature. 2024 May 15;629(8014):1126–32. doi: 10.1038/s41586-024-07424-x (PMC11136664; doi:10.1038/s41586-024-07424-x)
Supplement: Supplementary file 1 — Supplementary Tables 1 and 2 [file 41586_2024_7424_MOESM1_ESM.pdf]

nature portfolio

https://doi.org/10.1038/s41586-024-07424-x

Supplementary information

The temperature sensor TWA1 is required for thermotolerance in *Arabidopsis*

In the format provided by the authors and unedited

Supplementary Table 1. Primers used for genotyping and cloning.

PRIMER	5′→3′ sequence	REACTION
cpl3-10_f	TGGCGCCAATCACACGTCGCGCTGAC	G: *cpl3-10*
cpl3-10_r	CAACGAAGTACTCTCCAAAACACCAC	
jam1-2_f	ATGAATATGAGTGATTTAGGTTGGG	G: *jam1-2*
jam1-2_r	TTATATATCACCAGAGACCTGTG	
jam2-2_f	CTTTAGGCTCTCTGCAATATCCATCG	G: *jam2-2*
jam2-2_r	GAGAAAATCTCGGTGGAAGAGACTTG	
jam3-2_f	ATGGGTCAAAAGTTTTGGGAGAATC	G: *jam3-2*
jam3-2_r	TTACTGTGATAGAGAGGCAAGGAG	
tpl-8_f	TTGGTTCTCGCGAAAGATTAG	G: *tpl-8*
tpl-8_r	GCTATGCTGTTTATTGCAGGC	
tpr2-2_f	TCCGTCACTCGTGTAGCATGGAG	G: *tpr2-2*
tpr2-2_r	GTATCAAACTGAACAACTCCAGCTG	
tpr4-2_f	TTTGATTGTCGGGATTAGAGTAAAG	G: *tpr4-2*
tpr4-2_r	CCAACAGAAGCTGTAATCTTAGAGC	
GK-o8409_extended	ATATTGACCATCATACTCATTGCTGATCC	G: GABI-Kat lines
JMLBa1	TGGTTCACGTAGTGGGCCATCG	G: SALK lines
EcoRI-TWA1_f	CCGGAATTCATGTCTGGAAGCCAAGAGCCTAG	C: pGAD424
TWA1-BglII_r	GATCAGATCTTTAAAAACCATCACCACCGAGCTCATCCG	C: pGAD424
SmaI-C-JAM2_f	GATCCCCGGGCATGAATATTGGTCGCCTAGTGTGG	C: pGAD424
JAM2-SalI_r	GATCGTCGACCTATCTACCTGATGATGTTCTTGACTG	C: pGAD424
EcoRI-twa1-1_f	CCGGAATTCATGTCTGGAAGCCAAGAGCCTAG	C: pGAD424
twa1-1-BglII_r	GATCAGATCTTCAAACATCCATGGTAGTATTCAAATCCC	C: pGAD424
SmaI-Al-TWA1_f	TTGTCCCGGGTATGTCTGGAAGCCAAGAGCC	C: pGAD424
Al-TWA1-BglII_r	TTGTAGATCTTTAAAAACCATCACCACCA	C: pGAD424
SmaI-Sa-TWA1_f	TTGTCCCGGGTATGTCTGGAAACCAACAGCC	C: pGAD424
SalI-Sa-TWA1_r	TTGTGTCGACCTAAAACCCGTCACCACCACCA	C: pGAD424
SmaI-NotI-TPL-f	GATCCCCGGGGCGGCCGCATGTCTTCTCTTAGTAGAGA	C: pBRIDGE
TPL-NotI-SalI-r	GATCGTCGACGCGGCCGCTCATCTCTGAGGCTGATCAG	C: pBRIDGE
TPR1-BamHI_f	ATTAGGATCCCGATGTCTTCTCTGAGCAGAGAGC	C: pBRIDGE
TPR1-SalI_r	ATTAGTCGACTCATCTCTGAGGCTGGTCAG	C: pBRIDGE
EcoRI-TPR2-fwd	GATCGAATTCATGTCGTCTTTGAGCAGAGAG	C: pBRIDGE
TPR2-SalI_rev	GATCGTCGACTTACCTTTGAATCTGATCCGAACTTG	C: pBRIDGE
TPR3-BamHI_f	ATTAGGATCCCGATGTCGTCGTTGAGTCGAGAG	C: pBRIDGE
TPR3-SalI_r	ATTAGTCGACTCATCTTTGTAACTGTTCTGGAGTTTG	C: pBRIDGE
TPR4-BamHI_f	ATTAGGATCCCGATGTCGTCACTCAGCAGAGAAC	C: pBRIDGE
TPR4-SalI_r	ATTAGTCGACCTACGAATCACTCGGTTGTTGATCTG	C: pBRIDGE
EcoRI-JAM1_f	gatcGAATTCATGAATATGAGTGATTTAGGTTGGGATG	C: pBRIDGE
JAM1-SalI_r	GATCGTCGACTTATATATCACCAGAGACCTGTGA	C: pBRIDGE
BamHI-JAM2_f	GATCGGATCCATGAATATTGGTCGCCTAGTGTGG	C: pBRIDGE
JAM2-SalI_r	GATCGTCGACCTATCTACCTGATGATGTTCTTGACTG	C: pBRIDGE
SmaI-C-JAM3_fwd	GATCCCCGGGCATGGGTCAAAAGTTTTGGG	C: pBRIDGE
JAM3-BamHI_r	GATCGGATCCTTACTGTGATAGAGAGGCAAGGAGC	C: pBRIDGE

SmaI-C-MYC2_fwd	CCCGGGcATGACTGATTACCGGCTACAACC	C: pBRIDGE
MYC2-PstI_r	GATCCTGCAGTTAACCGATTTTTGAAATCAAACTTGCTC	C: pBRIDGE
Sal-AlHVR-TWA1_f	TTGTGTCGACATGTCTGGAAGCCAAGAGCCTAGG	C: pGREG574
TWA1-SalI_r	TTGTGTCGACTTAAAAACCATCACCACCGA	C: pGREG574
EcoRI-TWA1_f	CCGGAATTCATGTCTGGAAGCCAAGAGCCTAG	C: pSK_AscI_p35SS1-3xHA-tag-10xAla-nosTER
TWA1-BglII_r	GATCAGATCTTTAAAAACCATCACCACCGAGCTCATCCG	C: pSK_AscI_p35SS1-3xHA-tag-10xAla-nosTER
PstI-ATG-ΔN-TWA1_f	GATCCTGCAGATGGATGAACTTTCCAGAAATGATGAAGAG	C: pSK_AscI_p35SS1-nosTER
ΔN-TWA1-BglII_r	GATCAGATCTTTAAAAACCATCACCACCGAGCTCATCCG	C: pSK_AscI_p35SS1-nosTER
EcoRI-twa1-1_f	CCGGAATTCATGTCTGGAAGCCAAGAGCCTAG	C: pSK_AscI_p35SS1-nosTER
twa1-1-SalI_r	GATCGTCGACTCAAACATCCATGGTAGTATTCAAATCCC	C: pSK_AscI_p35SS1-nosTER
EcoRI-JAM1-f	GATCGAATTCATGAATATGAGTGATTTAGGTTGGG	C: pSK_AscI_p35SS1-nosTER
SalI-JAM1-r	gatcGTCGACTTATATATCACCAGAGACCTGTG	C: pSK_AscI_p35SS1-nosTER
SmaI-JAM2-f	GATCCCCGGGATGAATATTGGTCGCCTAGTGTGG	C: pSK_AscI_p35SS1-nosTER
JAM2-SalI_r	GATCGTCGACCTATCTACCTGATGATGTTCTTGACTG	C: pSK_AscI_p35SS1-nosTER
SmaI-C-JAM3_fwd	GATCCCCGGGCATGGGTCAAAAGTTTTGGG	C: pSK_AscI_p35SS1-nosTER
JAM3-BamHI_r	GATCGGATCCTTACTGTGATAGAGAGGCAAGGAGC	C: pSK_AscI_p35SS1-nosTER
SmaI-NotI-TPL-f	GATCCCCGGGGCGGCCGCATGTCTTCTCTTAGTAGAGA	C: pSK_AscI_p35SS1-nosTER
TPL-NotI-SalI-r	GATCGTCGACGCGGCCGCTCATCTCTGAGGCTGATCAG	C: pSK_AscI_p35SS1-nosTER
SmaI-NotI-TPR2-fwd	GATCCCCGGGGCGGCCGCATGTCGTCTTTGAGCAGAGAG	C: pSK_AscI_p35SS1-nosTER
TPR2-PstI-SalI-rev	GATCGTCGACCTGCAGTTACCTTTGAATCTGATCCGAACTTG	C: pSK_AscI_p35SS1-nosTER
SalI-TPR4_f	GATCGTCGACATGTCGTCACTCAGCAGAGAAC	C: pSK_AscI_p35SS1-nosTER
TPR4-BglII_r	GATCAGATCTCTACGAATCACTCGGTTGTTGATCTG	C: pSK_AscI_p35SS1-nosTER
SmaI-Al-TWA1_f	TTGTCCCGGGATGTCTGGAAGCCAAGAGCC	C: pSK_AscI_p35SS1-nosTER
Al-TWA1-SacI_r	TTGTGAGCTCTTAAAAACCATCACCACCA	C: pSK_AscI_p35SS1-nosTER
SmaI-Sa-TWA1_f	TTGTCCCGGGATGTCTGGAAACCAACAGCC	C: pSK_AscI_p35SS1-nosTER
SalI-Sa-TWA1_r	TTGTGTCGACCTAAAACCCGTCACCACCACCA	C: pSK_AscI_p35SS1-nosTER
pHSFA2-SacII_fwd	GATCCCGCGGCTTTGCCAATTCCTCTGTCCTCG	C: pSK_AscI_pHSFA2-LUC-nosTER
pHSFA2-BamHI_rev	GATCGGATCCTTCCACTTTCAGTTCTTCCATTTTCGTTG	C: pSK_AscI_pHSFA2-LUC-nosTER
BglII-3UTR-TWA1_f	GATCAGATCTAATAAAGGTTAGTATTCAATTTGCTTTAACCTTC	C: TWA1-3'UTR
3'UTR-TWA1-BglII_r	GATCAGATCTGAAAAGAAAAGACTATAAATGTGTGTAG	
NotI-pTWA1_f	GATCGCGGCCGCCTTTGTTCTTTCACTTCAACTCTTTGG	C: TWA1 promoter + TWA1-5'UTR
5'UTR-TWA1-SmaI_r	GATCCCCGGGAGTTGAACTTCGAGGGTCCTAAT	

Supplementary Table 2. Primers used for qPCR expression analysis.

PRIMER	5´→3´ sequence	REACTION	REFERENCE
TIP41L_f	GTGAAAACTGTTGGAGAGAAGCAA	qPCR reference gene (At4g34270)	
TIP41L_r	TCAACTGGATACCCTTTCGCA		
UBC9_f	TCACAATTTCCAAGGTGCTGC	qPCR reference gene (AT4G27960)	
UBC9_r	TCATCTGGGTTTGGATCCGT		
UBI10_f	GGCCTTGTATAATCCCTGATGAATAAG	qPCR reference gene (At4g05320)	
UBI10_r	AAAGAGATAACAGGAACGGAAACATAGT		
HSFA2_f	GTGTTGAGGTTGGGCAATACG	HSFA2 (AT2G26150)	Ayako et al, 2011 Plant and Cell Physiology, https://doi.org/10.1093/pcp/pcr045
HSFA2_r	TTGCTGTTGCCTCAACCTAACTAC		
HSP17.6_f	GCACGTGTTCAAGGCGGATTTAC	HSP17.6 (AT1G53540)	Volkov et al, 2003 Journal of Experimental Botany, https://doi.org/10.1093/jxb/erg244)
HSP17.6_r	TCACACATTTTTGGTCTTAAACAATTCC		
HSP18.2_f	CCGTTCTCGCAAGACTTATGG	HSP18.2 (AT5G59720)	
HSP18.2_r	GAAGCGTTTGCCAACGCAGAA		
HSP70	CATGACCGCGTCGAAATCATCG	HSP70 (AT3G12580)	Zhang et al 2019, Plant Signaling & Behavior,https://doi.org/10.1080/15592324.2019.1670596)
HSP70	GCCAGTGACTCTTATCCGCTTGAAC		
HSP21_f	CGCTTAACCATGGACGTCTCTC	HSP21 (AT4G27670)	Kim et al 2012, G3 Genes/Genomes/Genetics, https://doi.org/10.1534/g3.112.003368)
HSP21_r	CTGACACTCCACTTCCTCCTC		
HSP26.5_f	TGTGAAAGAGGTTTGGTCGG3	HSP26.5 (AT1G52560)	designed with Primer3Plus (bioinformatics.nl)
HSP26.5_r	TGTTACGCCAGAGGCTTTTT		
TWA1_qP	CTCCACTCAGATTCCACAACC	TWA1 (Atg513590)	
TWA1_qP	TCCCAATGCTCTTCCCTTTAC		
cis7_f	GACGTGGCAGGACGAAACGG	qPCR pRD29B promoter fragments, ChIP samples	
cis7_r	CTCTCTGGCTTCTGTCTCTTTAC		
cis6_f	CATCGATAGGCTTCTCTAAAGATC		
cis6_r	CAGCCAGTTGCTCTCTACGTG		
cis5_f	GCCAAATAACTACATGATGGGCC		
cis5_r	GATCTTTAGAGAAGCCTATCGATG		
cis4_f	GCTGAATTGATTTTTTCTTTTGCCG		
cis4_r	GCCCATCATGTAGTTATTTGGC		
cis3_f	GAGTACGTTATGCCGTTTTAAATG		
cis3_r	CGGCAAAAGAAAAAATCAATTCAGC		
